# Paraganglioma in pregnancy, a mimic of preeclampsia: a case report

**DOI:** 10.1186/s13256-023-03871-8

**Published:** 2023-04-07

**Authors:** Michelle D. Lundholm, Jessica Marquard, Pratibha PR Rao

**Affiliations:** 1 Department of Endocrinology, Diabetes and Metabolism Cleveland Clinic, 9500 Euclid Avenue, F20, Cleveland, OH 44195 USA; 2 Center for Personalized Genetic Healthcare, Genomic Medicine Institute, Cleveland Clinic, Cleveland, OH 44195 USA

**Keywords:** Paraganglioma, Pheochromocytoma, Pregnancy care, Hypertension, Case report

## Abstract

**Background:**

The new presentation of pheochromocytoma or paraganglioma in pregnancy is very rare and can be life-threatening for mother and child.

**Case presentation:**

We present the case of a 26-year-old gravida 3 para 2 otherwise healthy Caucasian woman at 34 weeks gestation who presented with new onset hypertension associated with headaches, dry heaves, diaphoresis, and palpitations. She was initially diagnosed with preeclampsia and treated with labetalol and an urgent cesarean section, delivering a healthy baby girl. The diagnosis of preeclampsia came into question when, 6 weeks postpartum, she continued to have hypertension with atypical features. Testing revealed metastatic paraganglioma associated with a succinate dehydrogenase B gene mutation. The patient was then started on alpha-adrenergic blockade and has had close blood pressure monitoring while discussion of advances therapies is ongoing.

**Conclusion:**

This case demonstrates how paraganglioma/pheochromocytoma can be misdiagnosed as preeclampsia due to the overlapping features of new-onset hypertension late in pregnancy accompanied by headache and proteinuria. It is impractical to routinely screen for paraganglioma/pheochromocytoma in all pregnant patients diagnosed with preeclampsia due to the rarity of these tumors and the harm from high false-positive rates. Therefore, it is incumbent on the provider to have a high degree of suspicion for paraganglioma/pheochromocytoma when clinical features are unusual for preeclampsia, such as intermittent palpitations, diaphoresis, orthostatic hypotension, or hyperglycemia. Early detection of paraganglioma/pheochromocytoma with interventions to mitigate the risk of hypertensive crisis greatly reduce maternal and fetal mortality. Fortunately, our patient delivered a healthy baby and did not have any additional pregnancy complications despite the delay in her diagnosis.

## Introduction

Pheochromocytoma (PCC) and paraganglioma (PGL) are neuroendocrine tumors originating from chromaffin cells capable of producing catecholamine hormones. These tumors are very rare, with an annual incidence of 2–8 cases per million people [1], of which 80–85% are PCC and the remainder are PGL [2]. Hypertension, either sustained or paroxysmal, is the most common symptom of a secretory chromaffin cell tumor, and approximately 0.2–0.6% of all cases of hypertension are attributed to PCC or PGL [3].

The incidence of PCC and PGL in pregnancy is very rare, estimated at 1 in 15,000–54,000 pregnancies [3]. Untreated PCC/PGL poses life-threatening risks to both mother and fetus. While catecholamines are enzymatically processed in the placenta, a catecholamine surge can vasoconstrict the uteroplacental circulation leading to uteroplacental insufficiency, placental abruption, and fetal demise [4, 5]. Hypertensive crises can lead to acute maternal cardiovascular and neurologic complications such as myocardial infarction, cardiomyopathy, arrhythmia, or stroke. Antenatal diagnosis of PCC/PGL is necessary to mitigate maternal mortality rates from 29% to 0% and fetal mortality rates from 29% to 12% [6]. However, PCC/PGL in pregnancy is readily mistaken for preeclampsia due to overlapping symptoms, and because the latter is over 600 times more common [7]. The early recognition of PCC/PGL in pregnancy requires a high index of suspicion in all hypertensive women to improve maternal and fetal outcomes.

We describe the case of a 26-year-old woman who presented at 34 weeks gestation with new-onset hypertension. She was diagnosed with preeclampsia, and metastatic PGL was only discovered in the subacute postpartum phase. We review important clues to distinguish PCC/PGL from preeclampsia, and how evaluation and management should be altered when chromaffin cell tumor is diagnosed in pregnancy.

## Case presentation

A 26-year-old Caucasian woman with no significant past medical history presented to the emergency department with a headache and nausea for 1 day. These symptoms were associated with new-onset hypertension up to 200/130 mmHg prior to arrival, dry heaves, diaphoresis, and palpitations. She was 34 weeks and 2 days pregnant with her third child (gravida 3 para 2) and her two prior pregnancies were uncomplicated. She did not have any family history of hypertension, malignancy, nor any endocrinopathies. Her only medications were a probiotic and prenatal vitamin. She denied any tobacco, alcohol, or other substance use. In the emergency room, she was afebrile with a heart rate of 97 beats/minute and blood pressure 164/122 mmHg. She was fully alert and oriented with regular tachycardia. Her lungs were clear and her uterus was gravid with a size appropriate for dates. She had a few reddened striae on her abdomen and no edema. Her serum electrolytes and renal function tests were unremarkable and her urine dipstick protein was over 100 mg/dL. She was diagnosed with preeclampsia with severe features and was admitted on scheduled labetalol, magnesium, and a nicardipine drip. She had an urgent cesarean section procedure without additional complications and delivered a healthy baby girl. The baby was observed in the neonatal intensive care unit for a week and then safely discharged home.

The diagnosis of preeclampsia came into question when, 6 weeks postpartum, the patient was still reporting blood pressure fluctuations from 110/70 to 200/130 mmHg. She continued to have intermittent frontal tension headaches, palpitations, and nausea. She had been on labetalol 100 mg twice daily for 6 weeks with minimal effect and was in the process of self-discontinuing the medication. Initial hormone investigation revealed thyroid stimulating hormone (TSH) 1.1 mIU/mL (0.270–4.200 mIU/mL), free thyroxine (FT_4_) 0.81 ng/dL (0.76–1.46 ng/dL), luteinizing hormone (LH) 1.79 mIU/mL (follicular 1.9–12.5 mIU/mL, luteal 0.5–16.9 mIU/mL), follicle-stimulating hormone (FSH) 6.0 mIU/mL (follicular 2.5–10.2 mIU/mL, luteal 1.5–9.1 mIU/mL), insulin-like growth factor 1 (IGF-1) 178 ng/mL (98–305 ng/mL), and prolactin 41.5 ng/mL (nonpregnant 2.8–29.2 ng/mL) in the setting of breast feeding. A random cortisol level in the afternoon was 23.8 μg/dL (PM 3.5–16.8 μg/dL) and a 24-hour urinary cortisol was 28.3 μg/day (< 45.0 μg/day). Her renin level was 19.9 pg/mL (upright 3.2–33.2 pg/mL) and aldosterone was 67.4 ng/dL (3.1–35.4 ng/mL), with a corresponding aldosterone-to-renin ratio of 3.4.

**Fig. 1:**
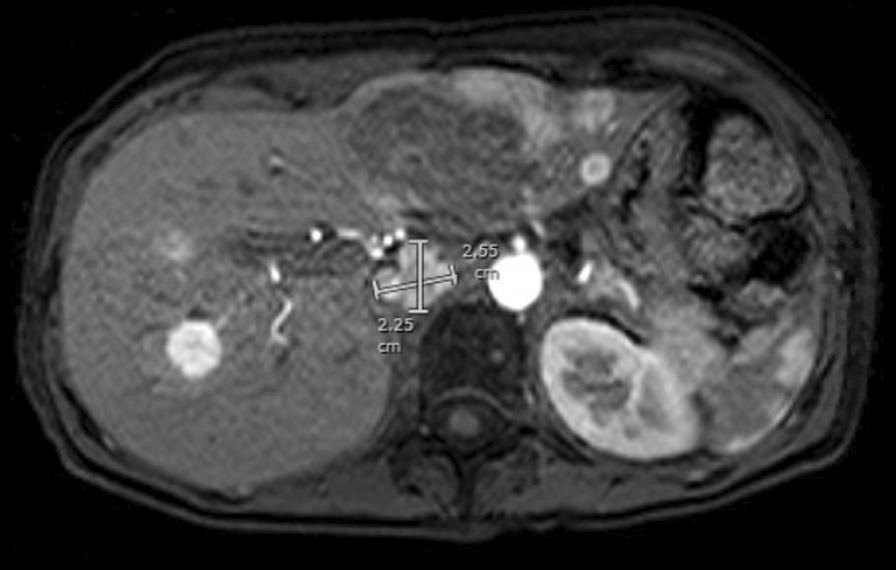
Magnetic resonance imaging of the abdomen with and without contrast. Highlighted is the largest retroperitoneal mass (2.65 × 2.25 cm)

**Fig. 2:**
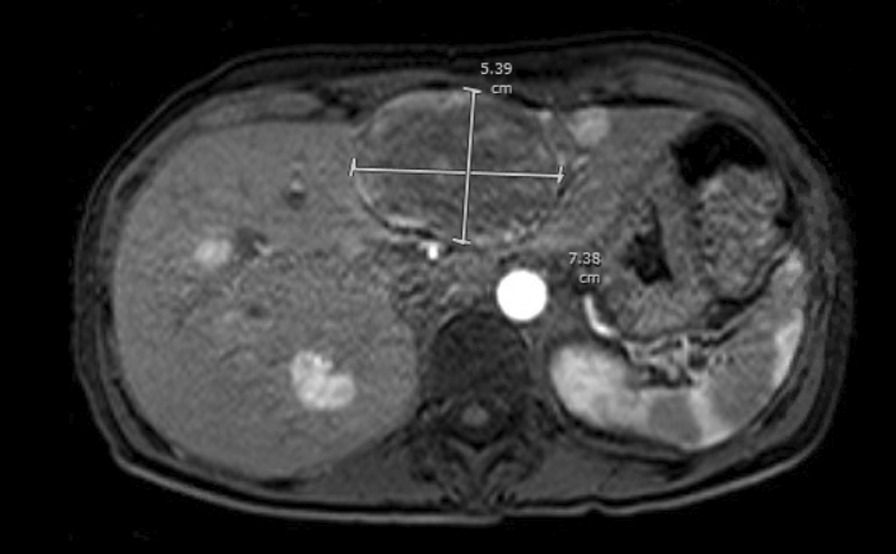
Magnetic resonance imaging of the abdomen with and without contrast. Highlighted is the largest hepatic mass (7.38 × 5.39 cm). Multiple additional enhancing hepatic masses are also seen

While awaiting the results of her outpatient workup, the patient returned to the emergency department with blurry vision, tinnitus, and headache, which were associated with a blood pressure of 182/110 mmHg. Additional blood work revealed a normal plasma metanephrine level of 48 pg/mL (12–67 pg/mL) and a significantly elevated plasma normetanephrine level of 19,950 pg/mL (18–101 pg/mL). Norepinephrine was also elevated to 13,752 pg/mL (80–520 pg/mL). Magnetic resonance imaging (MRI) of the abdomen showed multiple retroperitoneal para-aortic masses up to 2.6 cm in size with hypovascular enhancement, consistent with paragangliomas (Fig. 1). There were also multiple hepatic masses up to 7.4 cm in size with hypervascular enhancement, and multiple enhancing foci in the vertebral bodies, consistent with hepatic and osseous metastases (Fig. 2). This was later re-imaged with a 68-Ga-DOTATATE PET CT whole-body scan that showed multiple avid liver metastases, para-aortic retroperitoneal masses, and foci of uptake in the axial and appendicular skeleton.

She was diagnosed with a metastatic paraganglioma and her genetic testing was positive for a pathological variant (c.689G > A, p.Arg230His) in the succinate dehydrogenase complex subunit B (*SDHB*) gene. She was started on alpha-methyldopa in addition to labetalol and was referred to the endocrine surgery team. By consensus agreement between endocrine and surgical teams, she was recommended a laparoscopic debulking surgery for abdominal disease with liver and paraaortic lesion resection, with consideration of stereotactic body radiation therapy or cryoablation of bone lesions followed by a trial of 177-Lu-DOTATATE treatment. The patient opted for surveillance with follow-up appointments and blood work every 6 months and imaging every 6–12 months because she felt well and her blood pressure was well-controlled on her current medication regimen. Now, 3 years since her initial diagnosis, close monitoring and discussion of therapeutic options are ongoing.

## Discussion

This case demonstrates how readily a new case of PCC/PGL in pregnancy is missed, which presents providers an opportunity to improve maternofetal care with earlier PCC/PGL detection. A young woman at 34 weeks gestation presented with new-onset hypertension, headaches, palpitations, and diaphoresis and was misdiagnosed with preeclampsia with severe features. This patient and her child were fortunate to have a favorable outcome in a life-threatening situation: despite the potential for hypertensive crisis with beta-blockade therapy, she delivered a healthy baby girl by urgent cesarean section. The case uniquely continues with recognition of the missed diagnosis only weeks later when further hormonal and imaging workup discovered rare *SDHB*-related metastatic paraganglioma. We highlight significant clues in the presentation of PCC/PGL and how evaluation and management should be altered when these chromaffin cell tumors are diagnosed in pregnancy. With a high index of suspicion, early recognition in pregnancy can significantly mitigate maternal and fetal mortality risks.

Any woman with hypertension in pregnancy may be the 1 in 15,000 that has PCC/PGL; a thorough history, physical, and review of systems should be obtained in all patients to investigate. A meticulous personal and family history is necessary as up to 40% of PCC/PGL cases are related to germline mutations often associated with neurofibromatosis type 1, multiple endocrine neoplasia type 2, von Hippel–Lindau disease, and hereditary paraganglioma–pheochromocytoma syndrome [8].

Features of sustained hypertension, headaches, nausea, and proteinuria may be present in either preeclampsia or PCC/PGL, but symptoms such as paroxysmal hypertension, palpitations, diaphoresis, tremors, pallor, dyspnea, generalized weakness, orthostatic hypotension (due to hypovolemia and impaired vasoconstriction with postural change), and elevated blood glucose suggest potential PCC/PGL [9]. The symptoms of PCC/PGL often fluctuate with surges of catecholamine release, lasting for minutes to an hour at a time. Symptoms may also increase in frequency and intensity as pregnancy progresses; a gravid and growing uterus, fetal movements, and uterine contractions all have the potential to mechanically provoke tumor secretion [9, 10]. Our patient did not present with new-onset hypertension, diaphoresis, and palpitations until late into her third trimester of pregnancy.

Clinical judgment is needed to discern whom to screen for PCC/PGL given the costs associated with a high false-positive testing rate. Hypertensive women should be considered for screening if there is any personal or family history suggestive of a heritable PCC/PGL syndrome, or if her associated symptoms are atypical of preeclampsia. If indicated, the next step to evaluate for possible PCC/PGL is biochemical testing for plasma metanephrine or 24-hour urine metanephrine and catecholamine levels [11]. Pregnancy reference values have not been established, but it is worth noting that mild metanephrine and catecholamine elevations are commonly seen in normal pregnancy and preeclampsia. The false-positive result rate has not been determined in pregnancy, but false-positives occur in up to 25% of all cases [12, 13]. The false elevations are often due to interference from medications, increased sympathetic activity, or nonideal testing conditions. When the results are indeterminate, testing can be repeated in an ideal setting, where plasma samples are drawn after 30 minutes of supine rest with an indwelling intravenous cannula *in situ*. Clonidine suppression testing is frequently used for indeterminate metanephrine or catecholamine levels in the general population, but is contraindicated in pregnancy due to potential adverse effects.

When biochemical testing is consistent with a diagnosis of PCC/PGL, the next step is imaging. In the general population, computed tomography (CT) scans are widely used for anatomic assessment but are associated with radiation exposure on the order of 5–20 mSv [14]. Therefore, CT is not recommended in pregnancy as it exceeds the US Nuclear Regulation Commission recommended limitation on total fetal radiation exposure of less than 5 mSv. Many providers use abdominal ultrasound as an initial imaging step, as it is readily available and inexpensive, but this can be technically challenging to perform late in pregnancy. The preferred method of imaging is magnetic resonance imaging (MRI) with judicious use of gadolinium contrast, which does not appear to be associated with significant harm to the fetus [11, 15]. Since small radioactive materials may cross the placenta, functional imaging is not recommended in pregnancy.

The significant improvements in maternal and fetal mortality with antepartum recognition of PCC/PGL are attributed to adjustments in management to avoid catecholamine surge and hypertensive crises. Many common medication classes can trigger hypertensive emergency, including dopamine D_2_ receptor antagonists, tricyclic antidepressants, monoamine oxidase inhibitors, sympathomimetics, chemotherapy agents, opioid analgesics, and neuromuscular blockers [16, 17]. Beta-adrenergic receptor antagonists are used particularly often in the treatment of hypertension in pregnancy, but can cause unopposed alpha-1-adrenergic receptor stimulation leading to vasoconstriction. This is most often reported in nonselective blockers such as propranolol, timolol, nadolol, or sotalol. Labetalol is very commonly used in the management of preeclampsia and has both alpha- and beta-adrenergic receptor blocking effects, but in a 1:7 ratio which can still predispose to hypertensive crisis [18, 19]. For this reason, labetalol use alone is contraindicated in patients suspected of having PCC/PGL. All patients with PCC/PGL should be started on alpha-adrenergic blockade treatment first for a few days before starting beta-adrenergic blockade [20]. In pregnancy, phenoxybenzamine is disfavored as it crosses the placenta and risks hypotension and respiratory depression in the fetus [21]. Doxazosin crosses the placenta as well, but without known adverse events [22]. The dose of doxazosin can start at 2 mg per day and is up-titrated as guided by symptoms, with a suggested blood pressure target of 140/90 mmHg [23].

When it comes to fetal delivery, it is important that mothers with PCC/PGL seek care from an experienced medical team which includes obstetric, anesthetic, and neonatal providers. Active labor has the potential to precipitate a hypertensive crisis, and historical data have shown higher fetal mortality rates with vaginal delivery so it is recommended to deliver all women with PCC/PGL via cesarean section [10]. Some exception may be made for those who are stably pretreated with alpha-adrenergic blockade and adequate sodium and fluid intake.

If surgical tumor removal is feasible, the optimal timing is in the second trimester (prior to 24 weeks of gestation), 7–14 days after pretreatment with alpha-adrenergic blockers [3]. Alternatively, surgery can be postponed to the postpartum stage, potentially even soon after cesarean delivery.

Finally, nearly half of all PCC/PGL cases are associated with inherited genetic alterations [8]. Identification of specific mutations can clarify the risks of recurrent, metastatic, or associated disease, as well as the risks to family members. For these reasons, all patients with PCC/PGL should undergo genetic testing. Our patient was found to have a pathogenic variant in *SDHB*, which is a tumor suppressor gene responsible for the production of the succinate dehydrogenase enzyme in mitochondria. This mutation leads to an accumulation of succinate that ultimately promotes cell division and neovascularization and is found in up to half of people with metastatic PGL. *SDHB* is associated with PGL more often than PCC, with an increased risk of GI stromal tumor and renal clear cell carcinoma [24]. *SDHB* follows an autosomal dominant pattern of inheritance and 18–40% of patients will develop a tumor, most often diagnosed around 30 years of age [25]. Many patients who test positive have no family history of PGL/PCC. Biochemically, these tumors secrete norepinephrine and normetanephrine. Pathologic variants in *SDHB* are more often associated with extra-adrenal tumors and the malignancy rate is estimated at 65% (25–27). All first-degree relatives should be offered genetic testing. Surveillance options for carriers include imaging of the head, neck, chest, abdomen, and pelvis every 2–3 years with biochemical screening annually to begin around age 6–10 years. All female carriers should be screened for PCC/PGL biochemically and with imaging before each pregnancy to aid in antenatal diagnosis.

## Conclusion

A young woman in her third trimester of pregnancy presented with new-onset hypertension and was diagnosed with preeclampsia. The true cause of her symptoms, metastatic PGL, was only discovered in the postpartum period. This case exemplifies how PCC/PGL in pregnancy can mimic preeclampsia, resulting in high maternal and fetal risks enhanced by standard medical care such as the administration of routine medications and vaginal delivery. A high clinical suspicion is needed to identify this rare diagnosis and select appropriate patients for antenatal screening. With early identification, medical and surgical interventions can significantly mitigate both maternal and fetal mortality risks. Finally, genetic testing is indicated in all patients with confirmed PCC/PGL as it can provide prognostic information for both the patient and her family members.

## Data Availability

Not applicable.

## Abbreviations

PCC: Pheochromocytoma
PGL: Paraganglioma
SDHB: Succinate dehydrogenase B
ED: Emergency department
TSH: Thyroid stimulating hormone
FT_4_: Free thyroxine
LH: Luteinizing hormone
FSH: Follicle-stimulating hormone
IGF-1: Insulin-like growth factor 1
MRI: Magnetic resonance imaging
PET: Positron emission tomography
CT: Computed tomography
